# Postoperative kinetics of pentraxin 3 (PTX3) after congenital heart surgery with cardiopulmonary bypass in pediatric patients

**DOI:** 10.1186/s13741-022-00269-w

**Published:** 2022-08-22

**Authors:** Radoslaw Jaworski, Katarzyna Dzierzanowska-Fangrat, Renata Grzywa-Czuba, Andrzej Kansy

**Affiliations:** 1 Department of Anesthesiology and Intensive Care, Faculty of Medicine, Medical University of Gdansk, Gdansk, Poland; 2 Department of Clinical Microbiology and Immunology, The Children’s Memorial Health Institute, Warsaw, Poland; 3 Department of Cardiothoracic Surgery, The Children’s Memorial Health Institute, Warsaw, Poland

**Keywords:** Pentraxin, PTX3, Procalcitonin, C-reactive protein, Pediatric cardiac surgery, Cardiopulmonary bypass, Radoslaw Jaworski and Katarzyna Dzierzanowska-Fangrat have contributed equally to this work and share first authorship.

## Abstract

**Background:**

Pentraxins are inflammatory proteins and markers of acute-phase responses. They are divided into short and long subgroups based on the length of the N-terminal region. The most studied short pentraxin is the C-reactive protein (CRP), which is known to be expressed in various inflammatory conditions, including surgical procedures. On the other hand, much less is known about the kinetics of long pentraxin 3 (PTX3) in surgical patients, especially in the pediatric population.

The aim of this prospective study was to determine the early postoperative kinetics of PTX3 in relation to procalcitonin (PCT) and CRP levels in children undergoing congenital heart surgery with cardiopulmonary bypass (CPB).

**Methods:**

A total of 21 children (9 boys and 12 girls, mean age 12 months) were included in the study. Blood samples for determination of CRP, PCT, and PTX3 levels were collected before the surgery and then immediately after its completion (postoperative day 0, POD 0) and subsequently at POD 1, 2, and 3.

**Results:**

Serum PTX3 concentrations increased significantly between POD 0 and POD 1 (mean values were 12.2 and 72.4 ng/ml, respectively, *p*<0.001), decreased between POD 1 and POD 2 (mean values were 72.4 and 23.6 ng/ml, respectively, *p*<0.001), and normalized on POD 3 (the mean value was 1.2 ng/ml).

**Conclusions:**

PTX3 concentrations are markedly elevated during the first postoperative day. Under normal circumstances, PTX3 rises and falls quickly, and its second rise in the early postoperative period may be abnormal, however, further studies are necessary.

## Background

The pentraxins are a group of proteins involved in the innate immune response. They are divided into short and long subgroups based on the length of the N-terminal region (Vilahur & Badimon, 2015). The best-known representative of short pentraxins is the C-reactive protein (CRP), which is primarily expressed in the liver in response to inflammation. PTX3 was the first identified long pentraxin, which differed from CRP in terms of gene organization, protein oligomerization, and expression pattern (Daigo et al., 2014). It is a multifunctional protein with complex regulatory roles in inflammation, extracellular matrix organization, and remodeling (Doni et al., 2017). PTX3 is rapidly produced by endothelial cells, macrophages, myeloid cells, and dendritic cells after stimulation with cytokines (IL-1, TNF-α) and endotoxin (Doni et al., 2017; Giacomini et al., 2018; Cieślik & Hrycek, 2012; Zlibut et al., 2019). It may directly reflect the local tissue involvement in an inflammatory process (Muller et al., 2001; Oztan et al., 2019). In humans, PTX3 levels rise rapidly and dramatically in various pathological conditions of inflammatory or infectious origin, such as endotoxic shock and sepsis (Daigo et al., 2014).

In surgical patients, the monitoring of acute phase markers can be helpful as an early indicator of postoperative infection. However, as these markers increase as a result of the trauma caused by the surgical procedure itself, knowledge of their kinetics is crucial to distinguish a physiological inflammatory response from infection. This is especially important in young children with cardiac defects who are operated on with the use of cardiopulmonary bypass (CPB). CPB is associated with a systemic inflammatory response syndrome (SIRS) which makes the usual markers of infection such as CRP difficult to interpret (Jaworski et al., 2018). Although the kinetics of CRP and procalcitonin (PCT) are well understood in various surgical procedures, including pediatric cardiac surgery, virtually nothing is known about the dynamics of PTX3 in this setting.

The aim of this prospective study was to determine the early postoperative kinetics of pentraxin 3 (PTX3) in relation to PCT and CRP in children undergoing congenital heart defects surgery with CPB.

## Materials and methods

The prospective study was approved by the local ethics committee (No. 54/KBE/2019), complied with the tenets of the Helsinki Declaration, and was funded by a hospital grant (The Children’s Memorial Health Institute internal grant No. 266/19 and S184/2019). All parents of the operated children signed informed consent to participate in the study.

### Patient selection

The study involved children with congenital heart defects referred to our clinic for surgical correction with CPB. The inclusion criteria were as follows: planned surgical correction with CPB lasting no longer than 120 min, surgery without planned deep hypothermic circulatory arrest, absence of clinical signs of infection, or any organ dysfunction identified preoperatively in routine laboratory tests. Exclusion criteria included body weight less than 3 kg, uncompensated hypothyroidism, clinical signs of acute infection, leucocytosis, preoperative steroid treatment, and reoperative surgery.

### Perioperative procedures

All children underwent routine preoperative echocardiography, chest X-ray, electrocardiography, complete blood count, and biochemistry analysis. The standard antibiotic prophylaxis protocol used in the study consisted of cefazolin at a dose of 30 mg/kg administered intravenously 1 to 10 min before skin incision. An additional cefazolin dose of 15 mg/kg was administered for CPB priming. If the procedure lasted more than 4 h, intraoperative redosing of cefazolin was applied. After the surgery, cefazolin was continued every 6 h for 48 h. The surgery was performed with typical ascending aorta cannulation and direct cannulation of both main veins (vena cava superior and vena cava inferior) with the administration of heparin (30 IU/kg body weight). Hypothermia in the range of 28–34°C was applied during CPB, and cardiac arrest was performed with standard antegrade blood cardioplegia. All children were routinely monitored in the postoperative period.

Blood samples were collected preoperatively, immediately after the completion of surgery in the postoperative ward (postoperative day 0, POD 0) and on POD 1, 2, and 3 as part of routine laboratory tests got every morning on consecutive postoperative days and sent to a local laboratory. Serum CRP and PCT concentrations were measured using a turbidimetric and electrochemiluminescence immunoassay, respectively. Serum CRP and PCT concentrations were considered to be within the normal range provided they were below 5 and 0.5 ng/ml, respectively.

### PTX3 analysis

Serum PTX3 was measured using an enzyme-linked immunosorbent assay (ELISA) in accordance with the manufacturer’s instructions (BioVendor Human Pentraxin 3 ELISA), with a limit of detection of 22 pg/ml. Samples were diluted prior to the assay (with an expected highest 200-fold dilution) and tested in duplicate. Absorbance was measured in a Microplate Reader ELx800^TM^ (BioTek Instruments Inc., USA) using GEN5 software (BioTek Instruments Inc., USA).

### Statistical methods

The Kolmogorov–Smirnov and Shapiro–Wilk *W* tests were used to assess the normality of the data distribution. As no data conformed to a normal distribution, they were analyzed using the non-parametric Mann-Whitney *U* test. Pearson’s chi-squared test (goodness-of-fit) was used to analyze the association between independent variables. CRP, PTX3, and PCT concentrations on different postoperative days were compared using repeated measures ANOVA. Post hoc tests were performed using the Bonferroni correction. Pearson’s chi-squared test was used to describe differences between categorical variables, and in subgroups comprising less than 5 cases, Yates’ correction was applied. Differences were considered significant when *p*-values were below 0.05. In the case of continuous variables, the mean value with standard deviation and range were evaluated. Categorical variables were described in terms of the number and percentage of each subgroup. The respective values were rounded up to one decimal place. The statistical analysis was performed using statistical software (SPSS version 20.0, SPSS Inc., USA).

## Results

### Patient characteristics

A total of 21 children underwent surgery and were included in the study, including 9 boys (42.9%) and 12 girls (57.1%). The mean age of the patients was 12 months (SD=5.8; range 3.3–21), and the mean weight was 7.9 kg (SD=2; range 4.9–12). Detailed patient characteristics are shown in Table 1. No infections in early postoperative period in this group of patients were observed.

**Table 1:** Demographic and perioperative characteristics of the 21 patients included in the study

Parameters	Number (%) or mean (SD; range)
Age, months	12 (5.8; 3.3–21)
Weight, kg	7.9 (2; 4.9–12)
Operation time, minutes	150 (57; 95–360)
CPB time, minutes	85 (53; 30–240)
AoX time, minutes	44 (29; 13–113)
Operation type	
ASD (both I and II) closure VSD closure Other types (ToF correction, CAVSD correction, AoV plasty)	8 (38.1%) 9 (42.9%) 4 (19.1%)
Number of operations with the use of artificial implants	9 (42.9%)

*SD* standard deviation, *CPB* cardiopulmonary bypass, *AoX* aortic cross-clamp, *ASD* atrial septal defect, *VSD* ventricular septal defect, *ToF* tetralogy of Fallot, *CAVSD* complete atrioventricular septal defect, *AoV* aortic valve

### Kinetics of acute phase markers in the early postoperative period

**Fig. 1:**
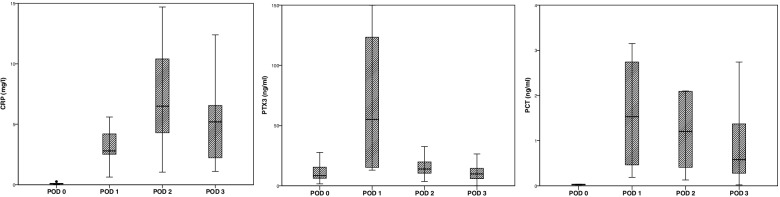
C-reactive protein (CRP), pentraxin 3 (PTX3), and procalcitonin (PCT) values in children immediately after surgery with cardiopulmonary (POD 0) and on postoperative days (POD) 1, 2, and 3

In all children, the preoperative CRP and PCT levels were within the normal range (< 5 mg/l and < 0.05 ng/ml, respectively). The preoperative mean PTX3 level was 4.6 ng/ml (SD=3.2; range 1.6–11.9 ng/ml). All patients exhibited elevated CRP, PTX3, and PCT levels in the early postoperative days, and these differences were statistically significant (Fig. 1; Table 2). The repeated measures ANOVA showed that mean CRP and PTX3 concentrations differed significantly between the operative day (POD 0) and POD 1, 2, and 3 (*F*=7.223 at *p*<0.001 and *F*=17.427 at *p*<0.001, respectively); these differences were not statistically significant for PCT. Post hoc tests (Bonferroni correction) revealed a significant increase in CRP concentration between POD 1 and POD 2 (mean CRP values were 3.1 and 6.9 mg/l, respectively, *p*=0.01), and a significant increase in PTX3 concentration between POD 0 and POD 1 (mean PTX3 values were 12.2 and 72.4 ng/ml, respectively, *p*<0.001) and a decrease in PTX3 between POD 1 and POD 2 (mean PTX3 values were 72.4 and 23.6 ng/ml, respectively, *p*<0.001). There were no significant changes observed for PCT in post hoc tests using Bonferroni correction. In all but two patients (90.5%), PTX3 decreased on POD 2 in comparison to POD 1.

**Table 2:** C-reactive protein (CRP), pentraxin 3 (PTX3), and procalcitonin (PCT) concentrations in children immediately after surgery with cardiopulmonary bypass and on postoperative days 1, 2, and 3

Parameter	Day of observation	Number of observations	Mean; median (SD; range)	***p***-value
C-reactive protein (mg/l)	Operation (POD 0)	5	0.11; 0.1 (0.09; 0.03-0.26)	<0.001
	POD 1	21	3.1; 2.8 (1.35; 0.63-5.6)	
	POD 2	21	6.9; 6.5 (3.8; 1-19.2)	
	POD 3	12	6.22; 5.2 (5.3; 1.1-19.2)	
Pentraxin 3 (ng/ml)	Operation (POD 0)	21	12.3; 8.5 (10.2; 1.6-43.8)	<0.001
	POD 1	21	72.4; 55.2 (57.4; 13-193.4)	
	POD 2	21	23.6; 14 (24.1; 3.6-97)	
	POD 3	20	11.6; 11 (6.7; 3.3-26.5)	
Procalcitonin (ng/ml)	Operation (POD 0)	16	0.06; 0.03 (0.02; 0.02-0.53)	<0.001
	POD 1	20	4.3; 1.5 (7.9; 0.2-33.3)	
	POD 2	21	4.1; 1.2 (10.1; 0.1-45.9)	
	POD 3	21	2; 0.6 (5; 0.02-23.1)	

*SD* standard deviation, *POD* postoperative day

## Discussion

In children undergoing surgery with the use of CPB, there are many uncertainties in the early postoperative days regarding infection status and appropriate antibiotic use. Although the recommendations for perioperative antibiotic prophylaxis in cardiac surgery are well known, several authors have reported that in many centers, especially pediatric ones, the antibiotic prophylaxis is commonly extended for more than 24–48 h in fear of possible infection that could complicate the postoperative course (Jaworski et al., 2019; Alphonso et al., 2007). The situation is even more complex in patients whose sternum is left open for several days after the surgery.

Therefore, clinicians need reliable tools to guide their decisions on antibiotic use in the early postoperative period to avoid their overuse without increasing the risk of infectious complications. One option is to monitor the concentrations of inflammatory markers. However, this requires precise knowledge of their kinetics in a specific group of patients, especially when interference with additional factors (such as, e.g., the use of CPB) may be significant. Currently, the most widely used acute phase marker is CRP, a short pentraxin produced during a systemic inflammatory response (Doni et al., 2017). However, due to its kinetics (slow rise and long time to normalization), it is of limited value in postoperative monitoring, and studies have shown that PCT performs better in this setting (Farias et al., 2021).

Recently, many authors described PTX3 as a potential biomarker of the acute phase in various clinical settings. Serum PTX3 levels were shown to correlate with progression of autoimmune diseases (childhood-onset systemic lupus erythematosus (Quismorio Jr. & Quismorio, 2018), juvenile idiopathic arthritis, atopic dermatitis (Marseglia et al., 2012)), degenerative disorders, arteritis (Hajishengallis & Russell, 2015), and a variety of cardiovascular diseases (Cieślik & Hrycek, 2012; Inoue et al., 2012), as well as acute pancreatitis (Gluszek et al., 2020) and appendicitis (Ates et al., 2020). Studies conducted in neonates revealed that PTX3 is a good biomarker of early-onset sepsis (Densen & Ram, 2015; Baumert et al., 2021). It has also been shown that PTX3 may play a role in tissue repair in various models of non-infectious tissue damage, such as excisional skin wounds, chemically induced liver, and lung injury or arterial thrombosis (Doni et al., 2017).

Normal plasma PTX3 levels in healthy individuals are ≤ 2 ng/ml; slightly higher concentrations are observed in females than in males; levels also increase with age (Ristagno et al., 2019; Garlanda et al., 2018; Peri et al., 2000; Yamasaki et al., 2009). However, normal plasma PTX3 concentrations in children have not been determined so far. The main characteristic of PTX3 is that it rises faster than CRP (peak levels after 6–8 h and 24–48 h post stimulation, respectively) (Ristagno et al., 2019; Garlanda et al., 2018). Patients with acute myocardial infarction show an early peak in PTX3 levels observed within 6–8 h of symptom onset to values 3–5 times the normal range. Baseline PTX3 levels are reached within 3 days after stimulation (Daigo et al., 2014; Peri et al., 2000; Helseth et al., 2014).

To the best of our knowledge, there are currently no studies on the kinetics of PTX3 in patients undergoing cardiac surgery with the use of CPB, especially in children. Cardiac surgery with CPB induces a non-specific acute inflammatory response, and the contact of cellular and humoral blood components with the synthetic material of CPB provokes SIRS. This causes activation of leukocytes and endothelial cells, leading to massive cytokine release.

In this study, we investigated the kinetics of PTX3 in children in the early period after surgery for congenital heart disease with the use of CPB. PTX3 concentrations were compared to other commonly used biomarkers, namely CRP and PCT. We observed that the peak value of PTX3 was reached within 24 h after the operation with CPB, with the mean peak value of 72.4 ng/ml (and a maximum value as high as 193.4 ng/ml). This was significantly higher than the expected reference range. In all but two patients (90.5%), PTX3 decreased on POD 2 in comparison to POD 1 and in this aspect, it was similar to the changes in PCT; however, the proportion of this reduction seemed to be different. The reduction in PCT was rather little, while the mean PTX3 concentration on POD 2 was only 30% of that on POD 1. Moreover, on POD 3, the PTX3 concentrations were in the reference range, while PCT remained elevated. Therefore, the kinetics of PTX3 appear more favorable than other acute phase markers in pediatric cardiac surgery patients in the early postoperative period; however, our results concern children without postoperative infections. The rapid decrease of PTX3 on POD 2 could be a positive prognostic factor for an uneventful postoperative course in terms of infection. However, larger clinical studies should be performed to verify these observations. In our opinion, it is very important because this is the time when antibiotics prophylaxis ends, and physicians have to decide whether to start empirical antibiotic treatment or not. This decision is twice as difficult because the CRP value on POD 2 is almost always higher than on POD 1. It is not uncommon to see high body temperature or even fever with tachycardia on POD 2 associated with SIRS in this group of patients. However, it is worth emphasizing that the decision on empirical antibiotic therapy in patients after CPB should not be based solely on CRP, PTX3, or PCT values.

### Limitations

Our study has some limitations. Firstly, it included a small number of patients. Secondly, we could not perform advanced pharmacokinetic analysis due to the limited number of blood samples that could be taken from children due to ethical concerns related to the pediatric population. Additionally, there are far more complex procedures in pediatric cardiac surgery than in our selected patient group; therefore, our results cannot be simply extrapolated to all groups of pediatric patients.

We demonstrated that PTX3 concentrations were significantly higher in the early postoperative days in children after congenital heart surgery with CPB, with maximum values at 24 h after the procedure. Under normal circumstances, PTX3 rises and falls quickly in contrast to CRP and PCT, and a second PTX3 rise in the early postoperative period may be abnormal; however, this needs further investigations. A single assessment of any biomarker in the early postoperative period in these patients may lead to overdiagnosis of infections and overuse of antibiotics. Patient status can be more reliably monitored using the dynamics of biomarker concentrations on consecutive postoperative days. Multicentre prospective clinical studies should be performed to confirm our observations.

## Data Availability

The datasets used and/or analyzed during the current study are available from the corresponding author on reasonable request.
